# Supplementary material for: Diversity and Molecular Barcoding of Stink Bugs (Hemiptera: Pentatomidae) Associated with Macadamia in South Africa
Source: Insects. 2022 Jun 30;13(7):601. doi: 10.3390/insects13070601 (PMC9317150; doi:10.3390/insects13070601)
Supplement: Supplementary file 1 [file insects-13-00601-s001.zip › insects-1752410-supplementary.pdf]

Table S1. Number of stink bug morphospecies found at each location across three seasons.

	2017-2018 season		2018-2019 season		2019-2020 season					
	Limpopo		Limpopo		Limpopo		KwaZulu-Natal		Mpumalanga	
Stink bug species	Farm 1	Farm2	Farm 1	Farm2	Farm 1	Farm2	Farm1	Farm2	Farm1	Farm 2
Agonoscelis versicoloratus	1		5				2			
Anolcus campestris			5				2			
Antestia sp.	1		8	1			1		16	
Antestiopsis thunbergii			2					4		
Aspavia albidomaculata								21		
Basicryptus costalis							3	1		
Bathycoelia distincta	11	347	100	531	327	173	1770	22	273	150
Boerias sp. 1							182	33		
Boerias sp. 2							91	23		
Carbula recurva	1		7				1	11		
Caura rufiventris		5	16			1				
Chinavia pallidoconspersa	2	6	11	1	9		13	5	72	13
Coenomorpha nervosa							6	1		1
Macrorhaphis acuta	1						2	1		
Nezara viridula	52	207	2	10	70	7	21	9	43	2
Parachinavia prunasis	1	24	2	9	138		10	2	34	
Parantestia sp.	23	40	24	16	68	2	34	37	39	7
Piezodorus sp.	25	21	2	2	140				26	
Platacantha lutea							1		1	3
Pseudatelus raptorius			5		1	2	46	7		3
Tripanda signitenens			3				3	4		
Total numbers	768		762		938		2369		683	
	5520									

Table S2. Seasonal presence of stink bug species per scout batch per region.

	2017-2018		2018-2019		2019-2020					
	Limpopo		Limpopo		Limpopo		KwaZulu-Natal		Mpumalanga	
Stink bug species	Early	Late	Early	Late	Early	Late	Early	Late	Early	Late
Agonoscelis versicoloratus	1			3			2			
Anolcus campestris				4			2			
Antestia sp.	1			6				1		2
Antestiopsis thunbergii				1						
Aspavia albidomaculata							1	8		
Basicryptus costalis								3		
Bathycoelia distincta	5	13		43	35	15	53	40	11	12
Boerias sp. 1							22	51		
Boerias sp. 2							13	24		
Carbula recurva	1			3			1	8		
Caura rufiventris	1			3	1					
Chinavia pallidoconspersa	1	4		7	5	1	9	5	5	11
Coenomorpha nervosa							3	5		
Macrorhaphis acuta	1						2	1		
Nezara viridula	13	17		11	26	5	14	7	2	12
Parachinavia prunasis	2	7		4	23		5	1	7	
Parantestia sp.	9	1		15	10		21	14	8	4
Piezodorus sp.	9	2		3	24		6		6	
Platacantha lutea							1			
Pseudatelus raptorius				2	2		10	14		
Tripanda signitenens				3			5	2		
Total scout batches	37		42		55		172		36	
	342									

Table S3. Collection details of specimens sequenced to determine species presence and composition of stink bugs in macadamia orchards in South Africa.

Sample code	Sub family	Tribe	Morphological identification	Date collected	Location	SANC voucher numbers
MSL67	Pentatominae	Agonoscelidini	*Agonoscelis versicoloratus* (Turton, 1802)	11/2/2019	Limpopo 1	PENT00039
MSL68				26/2/2019	Limpopo 1	PENT00006
MSL69	Pentatominae	Halyini	*Anolcus campestris* Bergroth, 1893	14/2/2019	Limpopo 1	PENT00040
MSL70				5/3/2019	Limpopo 1	PENT00032
MSM46	Pentatominae	Antestiini	*Antestia* sp. Stål, 1865	10/3/2020	Mpumalanga 1	PENT00041
MSL71				31/10/2017	Limpopo 1	PENT00035
MSK47	Pentatominae	Antestiini	*Antestiopsis thunbergii* (Gmelin, 1790)	25/09/2019	KwaZulu-Natal 1	PENT00042
MSK48				27/11/2019	KwaZulu-Natal 2	PENT00031
MSK49	Pentatominae	Eysarcorini	*Aspavia albidomaculata* (Stål, 1853)	10/4/2020	KwaZulu-Natal 2	PENT00034
MSK50				20/5/2020	KwaZulu-Natal 2	PENT00043
MSK51	Pentatominae	Phyllocephalinae	*Basicryptus costalis* (Germar, 1838)	21/4/2020	KwaZulu-Natal 1	PENT00044
MSK52				19/7/2019	KwaZulu-Natal 2	PENT00007
MSL72	Pentatominae	Bathycoeliini	*Bathycoelia distincta* Distant, 1878	26/11/2019	Limpopo 1	PENT00026
MSL73				31/10/2017	Limpopo 1	PENT00027
MSL74				31/10/2017	Limpopo 1	PENT00028

MSL75				18/04/18	Limpopo 2	PENT00029
MSK53				24/9/2019	KwaZulu-Natal 1	PENT00030
MSK54	Pentatominae	Cappaeni	*Boerias* sp. 1	11/7/2019	KwaZulu-Natal 1	PENT00011
MSK55				5/9/2019	KwaZulu-Natal 2	PENT00045
MSK56				13/7/2019	KwaZulu-Natal 1	PENT00046
MSK57				13/7/2019	KwaZulu-Natal 1	PENT00008
MSK58	Pentatominae	Cappaeni	*Boerias* sp. 2	16/1/2020	KwaZulu-Natal 1	PENT00012
MSK59				11/3/2020	KwaZulu-Natal 1	PENT00009
MSK60	Pentatominae	Eyasarcorini	*Carbula recurva* Distant, 1915	5/12/2019	KwaZulu-Natal 2	PENT00033
MSK61				15/4/2020	KwaZulu-Natal 2	PENT00047
MSL76	Pentatominae	Cappaeni	*Caura rufiventris* (Germar, 1838)	26/2/2019	Limpopo 1	PENT00048
MSL77				19/02/2019	Limpopo 1	PENT00013
MSK62	Pentatominae	Nezarini	*Chinavia pallidoconspersa* (Stål, 1858)	20/7/2019	KwaZulu-Natal 1	PENT00014
MSM47				11/5/2020	Mpumalanga 1	PENT00049
MSK63				3/6/2020	KwaZulu-Natal 2	PENT00050
MSL78				20/8/2019	Limpopo 1	PENT00051
MSK64	Pentatominae	Halyini	*Coenomorpha nervosa* Dallas, 1851	20/5/2020	KwaZulu-Natal 1	PENT00015
MSM48				12/3/2020	Mpumalanga 2	PENT00052
MSK65	Asopinae	-	*Macrorhaphis acuta* Dallas, 1851	12/6/2020	KwaZulu-Natal 1	PENT00018

MSL79				25/7/2017	Limpopo 1	PENT00053
MSM49	Pentatominae	Nezarini	*Nezara viridula* (Linnaeus, 1758)	11/5/2020	Mpumalanga 1	PENT00054
MSL80				04/2019	Limpopo 2	PENT00055
MSM50				12/3/2020	Mpumalanga 2	PENT00056
MSK66				10/7/2019	KwaZulu-Natal 2	PENT00057
MSL81				25/7/2017	Limpopo 1	PENT00020
MSL82				25/7/2017	Limpopo 1	PENT00019
MSM51	Pentatominae	Nezarini	*Parachinavia prunasis* (Dallas, 1851)	16/4/2020	Mpumalanga 1	PENT00036
MSM52				4/6/2020	Mpumalanga 1	PENT00037
MSL83				19/9/2017	Limpopo 1	PENT00058
MSL84				01/2019	Limpopo 2	PENT00059
MSL85	Pentatominae	Antestiini	*Parantestia* sp. (Linnavuori, 1973)	12/2/2019	Limpopo 1	PENT00025
MSL86				18/04/18	Limpopo 2	PENT00060
MSM53				20/06/06	Mpumalanga 1	PENT00061
MSK67				30/4/2020	KwaZulu-Natal 1	PENT00062
MSK68				20/3/2020	KwaZulu-Natal 2	PENT00063
MSL87	Pentatominae	Piezodorini	*Piezodorus* sp. (Fieber, 1860)	18/04/18	Limpopo 2	PENT00022
MSL88				5/9/2017	Limpopo 1	PENT00021
MSM54				6/6/2019	Mpumalanga 1	PENT00064

MSL89	Pentatominae	Eurysaspidini	*Platacantha lutea* (Westwood, 1837)	5/3/2019	Limpopo 1	PENT00065
MSM55				12/3/2020	Mpumalanga 2	PENT00023
MSK71	Pentatominae	Halyini	*Pseudatelus raptorius* (Germar, 1838)	24/4/2020	KwaZulu-Natal 1	PENT00024
MSK72				5/6/2020	KwaZulu-Natal 1	PENT00066
MSM56				12/3/2020	Mpumalanga 2	PENT00067
MSM57				12/3/2020	Mpumalanga 2	PENT00068
MSK73	Pentatominae	Cappaeni	*Tripanda signitenens* (Distant, 1898)	6/8/2019	KwaZulu-Natal 1	PENT00069
MSK74				6/3/2020	KwaZulu-Natal 2	PENT00038
